# Determination of Calcium and Phosphorus Digestibility of Individual Feed Ingredients as Affected by Limestone, in the Presence and Absence of Phytase in Broilers

**DOI:** 10.3390/ani14243603

**Published:** 2024-12-13

**Authors:** Kyle Marcus Venter, Roselina Angel, Jamie Fourie, Peter William Plumstead, Wenting Li, Henk Enting, Yueming Dersjant-Li, Christine Jansen van Rensburg

**Affiliations:** 1 Neuro Livestock Research, Kameeldrift, Brits 0250, South Africa; kyle@nlrhub.com (K.M.V.); jamie@nlrhub.com (J.F.); peter@nlrhub.com (P.W.P.); 2 Department of Animal Science, University of Pretoria, Pretoria 0002, South Africa; christinejvr@up.ac.za; 3 Department of Animal and Avian Sciences, University of Maryland, College Park, MD 20742, USA; 4 Danisco Animal Nutrition & Health (IFF), Wilmington, DE 19803, USA; wenting.li@iff.com; 5 Cargill Animal Nutrition and Health, Veilingweg 23, 5334 LD Velddriel, The Netherlands; henk_enting@cargill.com; 6 Danisco Animal Nutrition & Health (IFF), 2342 BH Oegstgeest, The Netherlands; yueming.dersjant-li@iff.com

**Keywords:** broiler, digestible calcium and digestible phosphorus, phytate phosphorus, plant-based ingredients

## Abstract

To begin formulating broiler diets on a digestible calcium (Ca) basis, robust Ca digestibility values for ingredients and factors affecting this digestibility are needed. This study had three main objectives: (1) determine the standardized ileal digestibility (SID) of Ca and phosphorus (P) for seven plant-based feed ingredients in broilers, (2) assess the impact of phytate source on SID Ca from limestone (LS), and (3) evaluate the effect of phytase on SID Ca and P for the different ingredients. Two experiments were conducted to satisfy these objectives. In Experiment 1, a 4 × 2 × 2 factorial design was used, with four plant-based feed ingredients (corn, wheat, sorghum, and full-fat soybean meal (FFS)), two LS inclusions in the diet (absence of LS and the inclusion of LS required to achieve 0.65% Ca in the final diet), and two phytase doses (0 and 1000 FTU/kg diet). Experiment 2 utilized a 3 × 2 × 2 factorial design with three plant-based ingredients (soybean meal (SBM), rapeseed meal (RSM), and sunflower meal (SFM)), two LS inclusions in the diet (absence of LS and the inclusion of LS required to achieve 0.65% Ca in the final diet), and two phytase doses (0 and 1000 FTU/kg diet). The trial had eight replicate pens (6 broilers/replicate) per treatment. Data were analyzed using a factorial analysis in JMP Pro 16.0 with means separation performed when *p* < 0.05, using Tukey HSD. The SID Ca in the absence of phytase for wheat (72.9%) and FFS (69.9%) was higher (*p* < 0.05) than for sorghum (54.5%) and corn (46.3%). In Experiment 2, the SID Ca in the absence of phytase from SFM (61.0%) was higher (*p* < 0.01) than RSM (42.7%) and SBM (46.8%). The SID Ca from added LS was affected by the ingredient, with diets containing wheat and FFS resulting in the lowest (*p* < 0.05) SID Ca versus those containing corn and sorghum irrespective of phytase dose in Experiment 1, and the lowest (*p* < 0.05) for SBM and RSM vs. SFM in the absence of phytase in Experiment 2. Phytase supplementation increased (*p* < 0.01) SID Ca and SID P across all feed ingredients compared to non-supplemented diets. There was a two-way interaction (*p* < 0.01) of LS addition and ingredient on SID P in both experiments. The results of this study provide SID Ca and SID P values from the selected ingredients and show that phytate from different ingredients reacts differently with Ca from LS and should be considered when developing SID coefficients of Ca and P for use in commercial broiler feed formulation. The SID coefficients of Ca and P for the individual feed ingredients evaluated in this study will allow for the further development and transition towards dCa and dP in commercial feed formulation.

## Simple Summary

Although total calcium (Ca) is still commonly used in commercial poultry diet formulation, it does not consider how much Ca the broilers can use on a biological level. This study aimed to measure how much Ca and phosphorus (P) from plant-based feed ingredients the broilers can digest, both with and without the addition of phytase. The goal of this study was to gather information to aid in the transition towards a system that uses digestible Ca and P for poultry diet formulation, improving accuracy, mineral utilization, and sustainability in global poultry farming.

## 1. Introduction

In modern broiler nutrition, calcium (Ca) remains the only macronutrient in commercial feed formulations for which requirements are specified based on total content rather than considering the digestibility or availability of the nutrient to the broiler [1,2]. This coupled with the slow adoption of a digestible phosphorus (dP) system leads to the potential over or undersupply of these nutrients to broilers. Most nutritionists utilize a total Ca (tCa) to available (AvP) or dP ratio to formulate for Ca and P without fully understanding the availability of Ca to the broiler from the ingredients used in the diet. Research has shown that optimizing Ca nutrition can minimize inorganic phosphate use by reducing phytate interactions with Ca particularly from limestone (LS) [3,4]. This underscores the need for standardized ileal digestible (SID) Ca values for ingredients, that would allow for reduced over or under formulation of Ca in broiler diets, and in doing so enhancing phytase efficacy and P and Ca digestibility. Furthermore, the development of SID Ca coefficients from plant-based feed ingredients allows for the formulation based on an actual digestible Ca (dCa) value that can allow for reduced tCa in the diets in turn reducing LS inclusion. Reduction in tCa has shown to lead to better amino acid [5], fat [6], phytase efficacy [7,8] and micro mineral [9] absorption, allowing for lower nutrient levels, representing for more economical and sustainable diets. The addition of LS is used as a predominant source of Ca in broiler diets due to its affordability and availability, accounting for 60–75% of the analyzable Ca in a typical grower diet. The variability in LS physical and chemical characteristics, such as solubility, particle size, mineral composition, and geological origin, significantly influences Ca digestibility in broilers [7,8,10,11,12,13,14,15,16]. Additionally, dietary phytate source, concentration as well as phytase concentrations further affect Ca digestibility through mineral phytate interactions [17,18]. Prediction equations to quantify these complex interactions influencing Ca digestibility from LS have been developed and validated [19]. The effect of phytate-P (PP) concentration on Ca digestibility from LS has been documented [18], however there is limited work evaluating the impact of phytate source on Ca digestibility from LS [8], where differences in Ca digestibility are noted based on the source of phytate in the diet.

The SID Ca values in published literature for individual feed ingredients demonstrate inconsistencies that can be attributed to differences in the methodology used to determine SID Ca [20]. Accurate Ca digestibility values for plant-based ingredients are essential for more accurate diet formulation and optimization of phytase efficacy. There is limited data available for Ca digestibility for feed ingredients of plant origin. Plant-based ingredients by nature have a lower Ca content compared to ingredients of animal origin, which has led to difficulty in determining the Ca digestibility as in the instance of corn (0.01 to 0.02% Ca). When determining Ca digestibility coefficients for feed ingredients with low Ca content (lowest in grains), it is essential to develop robust values due to the error associated with the determination of these ingredients. Even though Ca in corn is low, in a withdrawal diet with corn as the main grain, corn can account for 15 to 30% of the total analyzable Ca in the diet, underscoring the importance of determining Ca digestibility in this ingredient. In a typical commercial starter diet with an inclusion of 35% SBM with a Ca content of 0.24%, SBM can contribute 0.08% tCa or around 12% of the analyzable Ca in the diet. Feed ingredients that contribute significant amounts of Ca to the diet, such as meat and bone meals, monocalcium phosphate, and dicalcium phosphate, have been evaluated [8,21,22]. Currently, limited published values are available for the Ca digestibility from SBM [20,23] and few studies [23,24,25] are available for SID Ca from canola meals. There are no published values to the author’s knowledge for SID Ca for other ingredients of plant origin.

Phytase is commonly added to poultry diets to enhance P availability through its hydrolysis of phytate-bound P. However, its effects on Ca digestibility are less consistent, with studies reporting variable outcomes [26,27,28]. Increasing the dose of phytase, often exceeding the traditional 500 FTU/kg diet, has shown potential to improve growth performance and nutrient digestibility, including that of Ca and P, by reducing the anti-nutritive effects of phytate [29].

Given the complexities and inconsistencies in Ca and P digestibility across different feed ingredients and the potential effects on phytase efficacy, this study aims to determine the SID Ca and P from various feed ingredients with and without phytase supplementation as well as the impact that different feed ingredients have on SID Ca from LS. The findings will contribute to and further the development of more accurate broiler diet formulations, promoting better nutrient utilization, improving the economics of production [30] and reducing mineral excretion, which is important for the sustainability of poultry production.

## 2. Materials and Methods

The use of animals in the trial was approved by the University of Pretoria Animal Ethics Committee. A total of 1700 day-old Ross 308 male broilers were obtained from a local commercial hatchery located 1 h from the research site and placed in floor pens with artificial lighting in an environmentally controlled house. The broilers were fed a commercial type corn-soybean based pre-starter from placement to 8 days of age (0.90% Ca and 0.50% dP), starter (0.80% Ca and 0.40% dP) from 9 to 16 days of age and grower from 17 to 19 days of age (0.65% Ca and 0.28% dP), with all nutrients formulated to meet or exceed nutrient requirements of the broiler [1] with the exception for Ca and P which were formulated to meet but not exceed the broilers requirements [31,32]. Clean pine shavings (5 cm deep) were used as bedding during the rearing phase. House temperature was set to 34 °C at placement and lowered based on broiler’s comfort. The lighting schedule followed 24 h light (L) and 0 h dark (D) from hatch to day 3, followed by 14 L:10 D from day 4 to 7, 16 L:8 D from day 8 to 12, and 18 L:6 D for the remainder of the trial. On day 19, broilers were moved into battery cages (width × depth × height; 54 cm × 68 cm × 37 cm, respectively) that were preassigned to a treatment (TRT) following a randomized complete block design, with block included as a random effect. Broilers were weighed individually and selected to ensure similar initial weight and to minimize within pen broiler weight variation. Each cage contained six broilers, with the pen considered as a replicate, of which there were 8 per TRT. Each battery pen was equipped with a single nipple line (3 nipples) and a feeder trough. Broilers were checked twice daily, and the weight and cause of mortalities were recorded. Feed and water were offered for ad libitum consumption throughout the study.

### 2.1. Feeds and Treatments

The trial comprised two experiments to evaluate seven of the commonly used feed ingredients representing in excess of 90% of plant based feed ingredients used globally. Experiment 1 utilized a 4 × 2 × 2 factorial arrangement, with four different plant-based feed ingredients (corn, wheat, sorghum, and full-fat soybean meal (FFS)), two LS inclusions in the diet (no supplemental LS and inclusion of LS required to achieve 0.65% Ca in the final diet) and two levels of phytase (0 and 1000 FTU/kg diet). The phytase used was a novel-consensus bacterial 6-phytase variant expressed in *Trichoderma reesei* (Axtra^®^ PHY Gold, Danisco Animal Nutrition & Health, IFF, Oegstgeest, The Netherlands). All diets used in Experiment 1 were formulated to contain a PP concentration of 0.144% with the use of a basal diet (Table 1, with Ca, P, and PP formulated to 0.03% Ca, 0.7, and 0.05, respectively). Experiment 2 utilized a 3 × 2 × 2 factorial arrangement, using three differing plant-based feed ingredients (SBM, sunflower meal (SFM), and rapeseed meal (RSM)) two LS inclusions in the diet (no supplemental LS and inclusion of LS required to achieve the final diet of 0.65% Ca) with two levels of phytase (0 and 1000 FTU/kg diet). In both experiments, the diets with LS contained 0.65% Ca to ensure the diets were close to the birds requirements [1,31,32,33]. All diets from Experiment 2 were standardized to a PP concentration of 0.23% with the use of the same basal as in Experiment 1. There were also four additional diets included (Table 2), which were used to determine SID Ca and P from the ingredient and SID Ca from LS using the difference method outlined in Lemme et al. [34].

**Figure 1:**
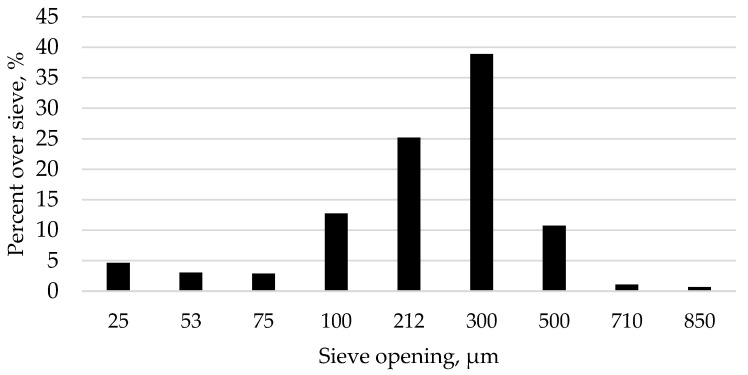
Particle size distribution of the limestone used in the study, determined based on the ANSI/ASAE method S319.4 [35].

**Figure 2:**
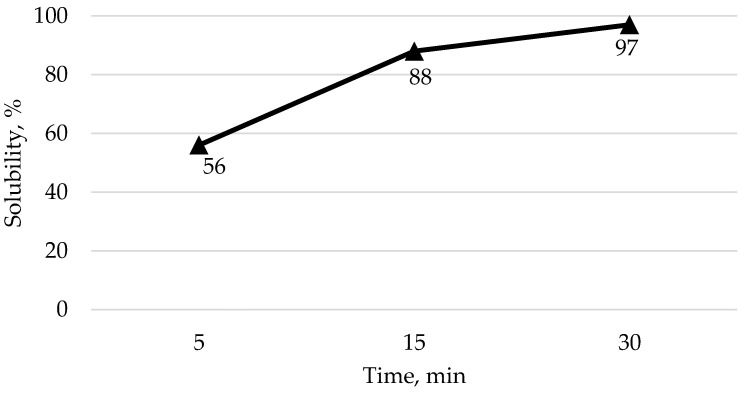
Dynamic solubility at 5, 15, and 30 min of the limestone used in the study, using a glycine-buffered pH 3 solution as per method of Kim et al. [16].

A commercial LS was obtained with a geometric mean diameter (GMD) of 509.1 µm (SD = 155 µm) (Figure 1) determined using ANSI/ASAE method S319.4 [35]. This LS had a solubility of 56, 88, and 97% at 5, 15, and 30 min, respectively (Figure 2) determined using a glycine-buffered pH 3 solution as per methodology in Kim et al. [16]. The LS was added after the diet was analyzed to achieve 0.65% Ca in all TRT diets except for the LS-free diets. Basal diet was mixed in a Hobart 20 kg planetary mixer. A previously prepared titanium dioxide (TiO_2_) mix, which contained 66.66% corn and 33.33% TiO_2_, ground three times through a 1.0 mm hammermill screen, was included in all final diets at 1.5%, contributing a total PP of 0.002% and TiO_2_ of 0.50% to the final diets. This mix was used to ensure a homogenous mix of the TiO_2_ into the final diet. The TiO_2_ was utilized as an undigestible marker to determine digestibility. When diets in the absence of LS were mixed, celite (Celite^®^, Minema, Johannesburg, South Africa) was added as an inert filler to achieve 100% of the final diet. All diets were tested with and without phytase. To do this, the diet, once mixed, was subdivided into two batches. Phytase was added to one of the batches based on the analyzed activity of the product (A novel consensus bacterial 6-phytase variant (Axtra^®^ PHY GOLD, Oegstgeest, The Netherlands (analyzed activity 42,820 FTU/g), Danisco Animal Nutrition & Health, IFF, The Netherlands), which was used to achieve 1000 FTU/kg diet, which aligns with common commercial practice. The diets were subsequently pelleted through a 3 mm die at a temperature reaching 74 °C at the exit of the pellet die, with representative samples taken for later analysis.

### 2.2. Ileal Sampling

Experimental diets were fed for 36 h to the broilers from day 19.5 to 21, following the methodology in Proszkowiec-Weglarz and Angel [36] and Tamim et al. [3]. At the end of 36 h, all broilers in a pen were euthanized using a 3 gas mixture of 35% CO_2_, 35%N_2_, and 30% O_2_, followed by CO_2_ asphyxiation [37]. The distal half of the ileum, from Meckel’s diverticulum to 3 cm above the ileocecal junction, was immediately removed. Digesta was flushed from the excised distal ileum using ice-cold deionized distilled water, and distal ileal content pooled by pen. The samples were then frozen at −20 °C and lyophilized. Lyophilized samples were ground using a mortar and pestle to pass through a 0.25 mm sieve and stored in airtight containers until further analysis.

### 2.3. Lab Analysis

The particle size of the LS was determined using the ANSI/ASAE method S319.4 [35]. Dynamic solubility of the LS was determined at 5, 15, and 30 min utilizing a pH 3 glycine buffered solution, in accordance with the methodology of Kim et al. [16]. All diets were ground to pass through a 0.5 mm screen prior to laboratory analysis, and samples were analyzed in duplicate.

Diet and digesta DM content were determined by drying for 24 h in a forced draft oven at 105 °C [38]). The Ca, P, and Ti content of the experimental diets and ileal digesta were determined after ashing and acid digestion using inductively coupled plasma atomic emission spectrometry [39]. Phytase activity was determined according to a modified version of AOAC method 2000.12, where one FTU was defined as the quantity of phytase that released 1 µmol of inorganic orthophosphate from a 0.0051 mol/L sodium phytate substrate per min at pH 5.5 at 37 °C [40].

### 2.4. Calculations

The determination of SID has its drawbacks based on the assumption of constant basal endogenous losses [41]. However, research has shown that the endogenous losses for Ca and P are impacted by diet type [42] but appear to be constant when methodology is standardized [36]. The *SID* of Ca and P were calculated based on the following formula using TiO_2_ as the inert marker:

(1) $$SID\;\left(\%\right)=\frac{{(Nutrient/{TiO}_{2})}_{diet}{-(Nutrient/{TiO}_{2})}_{ileal}}{{(Nutrient/{TiO}_{2})}_{diet}}\times 100-endogenous\;loss\;\left(\%\right)\;\;\;\;\;\;$$

Endogenous losses for Ca and P were determined as the mean obtained for eight studies being 106.8 (1.01 SE) mg/kg DMI and 146.29 (3.35 SE) mg/kg DMI, respectively [36].

dCa and dP were calculated as follows:

(2) $$Digestible\;Nutrient\left(\%\right)=\left(SID\;Nutrient\right)\times {Nutrient}_{diet}\;$$

where *Nutrient_diet_* is the analyzed total Ca or P concentration of the feed ingredient.

The digestibility was determined using the difference method [34]. The *SID* of Ca from LS was calculated based on the following formulas:

(3) $${Digestible\;Ca}_{diet}=\left({Digestible\;Ca}_{LS}\right)+({Digestible\;Ca}_{LS\;free\;diet})$$

(4) $$SID\;{Ca}_{LS}=\frac{{Digestible\;Ca}_{LS}}{{Ca}_{LS}}$$

The SID of Ca or P from the ingredient was calculated based on the following formulas:

(5) $$\begin{array}{c} {Digestible\;Nutrient}_{Diet}\\ \;\;\;\;\;\;=\left({Digestible\;Nutrient}_{Basal}\right)\times Inclusion+\left({Digestible\;Nutrient}_{Ing}\right)\times Inclusion \end{array}$$

(6) $$SID\;{Nutrient}_{Ing}=\frac{{Digestible\;Nutrient}_{Ing}}{{Nutrient}_{Ing}}$$

where *Nutrient_Diet_* is the analyzed total Ca or P concentration of the feed ingredient.

### 2.5. Statistical Analysis

Data were analyzed using the fit model platform in JMP 16.0 [43]. The basal diets were used for the calculation of SID Ca from feed ingredients as well as LS and SID P from the ingredient. Where the SID Ca from the ingredient and LS was determined, the effect of LS was not included in any model for Ca, as the ingredient SID Ca was determined in the absence of LS, and the SID Ca from LS was determined using the diet supplemented with LS. A factorial analysis was conducted for both experiments to determine the main effects and their interactions. Tukey HSD [44] separation was conducted when an effect was significant. Significance was accepted at *p* < 0.05.

## 3. Results

The analyzed dietary values for Experiment 1 (Table 2, Table 3 and Table 4) and Experiment 2 (Table 2, Table 5 and Table 6) were close to those formulated for Ca, P, PP, and phytase. For both experiments, phytase activities in non-phytase diets were all below 100 FTU/kg diet, the detection limit of the assay, demonstrating that no cross-contamination occurred during the mixing of the TRT diets between phytase and non-phytase diets (Table 5 and Table 6). No differences in mortality and growth parameters were noted between treatments in both experiments.

The results in Table 7 summarize the SID Ca and P in the four basal diets in the presence and absence of LS and phytase. The diets (TRT 1–4) were included in both experiments to allow for the calculation of the SID Ca and P of the ingredients and the SID Ca of the LS using the difference method (Equations (3)–(6)).

### 3.1. Experiment 1

The ingredients SID Ca and P and LS SID Ca in the presence of different ingredients from Experiment 1 are presented in Table 8. For SID Ca of the ingredients there was an ingredient × phytase interaction (*p* < 0.05). However, for SID Ca from LS, there was a main effect (*p* < 0.05) of ingredient and phytase dose. When evaluating the response in SID Ca from ingredients, there was an ingredient effect (*p* < 0.05), with corn and sorghum showing similar (*p* > 0.05) SID Ca in the absence of phytase (46.40% and 54.44%, respectively). Corn had the lowest (*p* < 0.01) SID Ca of all feed ingredients evaluated in the absence of phytase (46.40%). A higher (*p* < 0.001) SID Ca was observed in the feed ingredients such as wheat and FFS compared to corn and sorghum in the absence of phytase (72.87% and 69.85% vs. 46.40% and 54.44%, respectively). An improvement (*p* < 0.001) in SID Ca with phytase supplementation was noted for corn, wheat, and sorghum (46.40% to 69.81%, 72.87% to 87.32%, and 54.44% to 77.57%, respectively). However, this improvement in SID Ca with phytase supplementation was not noted for FFS.

There was no interaction for SID Ca from LS in the present study; however, there was a main effect (*p* < 0.001) of ingredient on SID Ca from LS. An improvement (*p* < 0.05) in SID Ca from LS was observed when diets containing primarily sorghum were fed compared to those with wheat and FFS. An improvement (*p* < 0.05) in SID Ca from LS was also noted irrespective of phytate source when diets were supplemented with phytase (64.78% vs. 71.09%).

A three-way interaction (*p* < 0.01) was present for SID P between ingredient, LS, and phytase supplementation. There was a reduction (*p* < 0.01) in SID P when LS was added to the diet in the absence of phytase for corn, wheat, FFS, and sorghum (74.92% to 7.93%, 69.79% to 33.32%, 78.85% to 46.54%, and 78.36% to 42.97%, respectively), with the extent of the effect of LS varying by ingredient, resulting in the interaction seen. The lowest (*p* < 0.01) SID P was noted for corn with the addition of LS in the absence of phytase (7.93%). When phytase was added to diets containing LS, there was an improvement (*p* < 0.01) in SID P for all feed ingredients evaluated. Phytase addition was able to overcome the detrimental effect of LS on SID P in wheat, FFS, and sorghum diets, where no difference (*p* > 0.05) in SID P was noted between diets without LS or phytase and diets supplemented with both LS and phytase. The only exception was when diets contained corn, where there was a reduction (*p* < 0.01) in SID P in the diet with LS and phytase compared to the diet without LS or phytase (64.49% vs. 74.92%). However, in corn diets, phytase increased (*p* < 0.05) SID P from 7.93 to 64.49%, showing a 56.56% improvement. In the absence of LS, phytase improved (*p* < 0.05) SID P for all four ingredients tested.

### 3.2. Experiment 2

Table 9 summarizes the SID Ca and P from ingredients generally used as protein sources and SID Ca from LS in the presence of these different feed ingredients. An interaction (*p* < 0.05) was present for all parameters measured. When evaluating the response in SID Ca from various feed ingredients, there was an ingredient effect (*p* < 0.05), with SBM and RSM having similar SID Ca in the absence of phytase (46.80% and 42.65%, respectively). The lowest (*p* < 0.01) SID Ca in the absence of phytase was for RSM (42.65%). The SID Ca was higher in SFM compared to RSM and SBM in the absence of phytase (60.99% vs. 42.65% and 46.80%, respectively). An improvement (*p* < 0.001) in SID Ca from ingredients with phytase supplementation was noted for SBM and RSM (46.80% to 67.77% and 42.65% to 62.32%, respectively). However, this improvement in SID Ca with phytase supplementation was not noted for SFM. An interaction (*p* < 0.001) between ingredient and phytase was present for SID Ca from LS. The SID Ca from LS in diets without phytase supplementation was lowest (*p* < 0.001) in diets comprising SBM and RSM (37.76% and 39.73%) compared to SFM (52.51%). Supplementation of phytase improved (*p* < 0.05) SID Ca from LS for all three ingredients tested. However, when diets were supplemented with phytase at 1000 FTU/kg diet, the differences (*p* > 0.05) in SID Ca from LS between different feed ingredients was no longer noted leading to the interaction effect observed.

A three-way interaction (*p* < 0.001) was present for SID P between ingredient, LS, and phytase supplementation. There was a reduction (*p* < 0.001) in SID P when LS was added to the diets compared to when no LS was added, in the absence of phytase, for SBM, RSM, and SFM (81.59% to 49.11%, 57.88% to 31.99%, and 74.93% to 33.75%, respectively). The lowest (*p* < 0.001) SID P was noted for RSM and SFM with LS addition and no phytase supplementation. When phytase was added to diets with LS, there was an improvement (*p* < 0.01) in SID P for all ingredients evaluated. The addition of phytase was able to overcome the detrimental effect of LS on SID P in SBM and RSM, where no difference (*p* > 0.05) in SID P was noted between diets without LS or phytase (0 FTU/kg diet) as compared with diets supplemented with both LS and phytase (1000 FTU/kg diet). The only exception was SFM, where there was a reduction (*p* < 0.001) in SID P from the diet with LS and 0 FTU/kg diet compared to the diet with LS and 1000 FTU/kg of diet (74.93% vs. 65.69%). However, phytase improved (*p* < 0.05) SID P in the absence of LS for all feed ingredients.

## 4. Discussion

To date, broiler feed formulations and requirements do not consider the availability of Ca. Based on the findings of this study and others [7,8,10,11,12,14,15,16,22,23,24], it is evident that this approach needs to be reevaluated. The present study demonstrated differences in the SID Ca and P between various feed ingredients. Despite the importance of understanding SID Ca from different feed ingredients, research on SID Ca from plant-based feed ingredients is limited. There are some predetermined SID Ca values for corn, SBM, and canola; however, there is significant variation between these values, mainly being driven by differences in methodology [24,45].

The SID Ca from various feed ingredients determined in this study, such as corn (46.4%), are similar to those of earlier research and were within the range of 12 samples [20] and close to the assumed value of 50%, which did not include any animal experimental work from David [46]. When evaluating the SID Ca from SBM, there was similarity with some prior research (46.8% vs. 49% [20]); however, slight differences were observed compared to that reported by other authors (54.0% [23]). The SID Ca for other ingredients, such as RSM, however, varied considerably from previously determined values of 31% [24]; 17.8% [25]; and 53 at day 21 and 22% at day 42, respectively [23] compared to that determined in this study (42.7% at 21 days). In another study [23], where the SID Ca from SBM and RSM were determined to be higher than in the present study, the source of these differences is in large part due to methodology. The diets in that study contained very low Ca concentrations (less than 0.2% Ca), which is deficient at 21 and 42 days and were fed for 72 h, allowing for adaptation [36] to a deficiency, which results in an upregulation of Ca digestibility [36,47] within 40 h after the deficient diet is fed. Conversely, the lower SID Ca noted in [24] for RSM was determined in an experiment where the birds were fed diets in excess of Ca requirements for birds 24 days of age (0.90%) for 72 h, allowing the birds to adapt to the excess Ca [36,47], resulting in a down-regulation of Ca absorption. There are no other known SID Ca values for the other ingredients determined in this study. The values for SID P from ingredients in this study are similar to those in published tables such as CVB [48] for ingredients such as SBM, wheat, FFS, RSM, and SFM. However, differences were noted when comparing the SID P from corn and sorghum. This may in part be due to the very low Ca content in these grains as well as factors that affect SID P, such as Ca content [5,49], PP, and total P content [4], LS source [8,16], leading to the potential differences in SID P noted for these feed ingredients across published tables.

The improvements seen in this study due to the inclusion of phytase on SID Ca from both LS and various feed ingredients (except for FFS and SFM) highlight its crucial role in improving Ca availability in broiler diets. The inconsistent benefit of phytase for FFS and SFM may be pertaining to the nutrient composition or presence of antinutritional factors affecting the response. Extensive work on the impact of phytase on Ca digestibility has shown that phytase increases SID Ca by hydrolyzing phosphates from PP and that the affinity of lower inositol phosphates for Ca ions is reduced, releasing chelated Ca ions [5,49,50,51,52,53]. However, the degree and impact of phytase on Ca digestibility have been variable [8,26,27,28,54]. There are several factors that affect Ca digestibility, such as intrinsic digestibility of Ca from different raw materials, concentration of Ca in the diet [49,55,56], age of the animal [56], and nPP or phytate concentrations [57,58] could contribute to the inconsistencies seen in dietary Ca digestibility and phytase response reported in the literature. This work highlights the different interactions between the phytic acid in the ingredient and LS Ca and the effects on Ca made available by phytase.

Phosphorus from phytate has generally been considered to have a low availability in monogastric animals. Results from this study showed that when LS is not added, the P digestibility can be as high as 81.59%. Prior work has shown similar results [3,59]. The interaction between PP and Ca from LS is pivotal in determining the availability of P from phytate in ingredients and diets. This interaction and the subsequent detrimental effect on SID P have been documented extensively in prior research [5,49] and were noted in this study, where the addition of LS resulted in a reduced SID P in all feed ingredients in the absence of phytase. This highlights the inhibitory effects of Ca from LS on P digestibility in broilers. Due to this interaction between Ca and phytate being substrate driven, excess Ca or excess phytate can exacerbate this effect. There has been extensive work evaluating the impact of PP concentration on Ca and P digestibility [3,8,18]. Due to the saturable nature of phytate, high phytate concentrations are known to increase the formation of insoluble complexes with Ca, significantly reducing the digestibility of Ca and P [16,58]. However, supplementation of a phytase that is highly active in the upper gastrointestinal tract and that is added at a high concentration can hydrolyze most of the phytate prior to the chelation with Ca, minimizing this negative impact and enhancing overall nutrient utilization [59,60]. In this context, the addition of phytase is crucial to mitigate the negative impact of LS Ca, especially when LS has high solubility in the acid stomach [8] on Ca and P absorption. Studies have shown that higher doses of phytase (e.g., 1000 FTU/kg diet) are more effective in breaking down phytate complexes, thus improving Ca and P digestibility [17,61,62,63,64,65,66]. In the current study, phytase supplementation not only improved Ca digestibility but also improved P digestibility, making P digestibility similar to that of diets without LS addition. This likely occurred because phytase reduced the formation of insoluble Ca–phytate complexes through the hydrolysis of phytate prior to the chelation of Ca and phytate, thereby facilitating better P absorption [57,67]. Understanding SID Ca of ingredients and of LS Ca can result in better choice of LS and reduced concentrations of Ca in the diet. The reduction in dietary Ca has been seen as a potential strategy to reduce these Ca and phytate interactions and, in doing so, further improve P digestibility [68]. The findings of this study highlight the benefit of phytase and its importance in poultry nutrition, particularly in formulating diets to maximize nutrient digestibility.

The impact of phytate concentration on Ca and P digestibility is well documented [18,57,59]. However, limited studies have evaluated if phytate source influences Ca digestibility. The impact of phytate source on Ca and P digestibility, as observed in this study, aligns with prior findings [8,68]. These studies showed that corn-derived phytate had a large adverse effect on SID Ca and P due to its high reactivity with Ca, leading to the formation of insoluble Ca-phytate complexes in the gastric area of the gastrointestinal tract, thus reducing the beneficial effects of phytase [3,60]. This may be explained in part by cereal-derived phytate having a higher reactivity toward divalent cations than protein-source phytate because it is stored as free or loosely bound molecules, exposing reactive phosphate groups that readily chelate minerals like calcium and magnesium, particularly in acidic conditions [51]. In contrast, phytate in protein sources is tightly bound to proteins, forming stable complexes that reduce its ability to interact with minerals, limiting chelation and mineral bioavailability relative to cereal grains [69,70]. In the current study, corn had the lowest SID P, even with the addition of 1000 FTU/kg of diet. This amount of phytase was unable to increase the digestibility of P to that of the same diets without LS added. This is consistent with previous findings that the phytate digestibility in corn remains low in the presence of phytase [8,68]. Li et al. [8] observed that the effectiveness of phytase in improving Ca and P digestibility varied with the source and concentration of dietary phytate. High concentrations of phytase (1000 FTU/kg diet) were more effective in counteracting the negative effects of diets high in phytate originating from corn. Phytase addition in this study was able to increase P digestibility in diets with wheat, sorghum, and FFS back to digestibility’s of P seen in diets with the same ingredients but without LS addition. In Experiment 2, SFM had the lowest P digestibility in the absence of phytase, and phytase was unable to take P digestibility to the level seen in the diet without LS. This suggests that similar to corn, SFM exhibits a phytate that is more interactive with ionized Ca from LS, leading to a phytate–Ca chelation that is less susceptible to the action of phytase. To the authors’ knowledge, there is no existing research in vivo documenting these interactions. The primary limitation is that, in certain cases, only a single value for SID Ca is available, which may compromise the robustness. Further research is required to evaluate the variability of SID Ca within individual feed ingredients. Preliminary findings [30] indicate that when diets are formulated using ingredient-specific SID Ca values and fed to broilers, the resulting dietary SID Ca levels closely align with the formulated targets.

## 5. Conclusions

In conclusion, several key points emerge from this study. First, the digestibility of Ca should be considered in feed formulation, given the considerable differences in Ca digestibility among different feed ingredients. Second, the interaction between Ca and phytate, as well as the efficacy of phytase, is influenced by the sources of phytate and by LS addition. Therefore, establishing robust reference values for SID Ca in plant-based feed ingredients and understanding the factors affecting SID Ca from LS are essential. This knowledge is critical for advancing a dCa and dP system, ensuring more precision when formulating diets to maximize nutrient digestibility and, in doing so, minimize environmental mineral excretion. More research on ingredient dCa values is needed, but it will be important to have these values generated with the same methodology. Adoption into industry will require shadow formulation with dCa values for feed ingredients, and preliminary research into commercial diets so far shows improvements in performance and economics.

## Figures and Tables

**Table 1:** Low calcium and phytate phosphorus basal diet formulation.

Ingredient, %	
Degermed corn, 6.5% CP	45.99
Rice ^1^, 7.3% CP	45.99
Hemoglobin ^2^, 90% CP	4.99
Soybean oil	3.03
Total	100.00
Formulated (analyzed) values	
Total Ca	0.03 (0.03)
Total P	0.07 (0.10)
Phytate P	0.05 (0.10)

^1^ Polished jasmine rice. ^2^ Porcine hemoglobin.

**Table 2:** Formulation for diets used to calculate SID Ca from limestone (LS) and from the feed ingredients.

Ingredient, %	Treatment 1 ^1^	Treatment 2 ^2^	Treatment 3 ^3^	Treatment 4 ^4^
LS ^5^	−	+	−	+
Phytase ^6^, FTU/kg diet	0	0	1000	1000
Basal diet ^7^	95.96	95.96	95.96	95.96
Limestone	0.00	1.63	0.00	1.63
Celite ^8^	1.63	0.00	1.63	0.00
Vitamin premix ^9^	0.15	0.15	0.15	0.15
Mineral premix ^10^	0.15	0.15	0.15	0.15
Salt	0.20	0.20	0.20	0.20
Sodium bicarbonate	0.20	0.20	0.20	0.20
Choline chloride, 60%	0.20	0.20	0.20	0.20
Xylanase ^11^	0.01	0.01	0.01	0.01
TiO_2_ blend ^12^	1.50	1.50	1.50	1.50
Phytase	0.00	0.00	0.003	0.003
Total	100.00	100.00	100.00	100.00
Analyzed (formulated) values				
Total Ca	0.03 (0.03)	0.65 (0.63)	0.03 (0.03)	0.65 (0.63)
Total P	0.07 (0.09)	0.07 (0.09)	0.07 (0.09)	0.07 (0.09)
Phytate P ^13^	0.05 (0.10)	0.05 (0.08)	0.05 (0.10)	0.05 (0.08)

^1^ Used in the calculation of SID Ca from the feed ingredient in the absence of phytase using the difference method. ^2^ Used in the calculation of SID Ca from the limestone and SID P from the ingredient in the absence of phytase using the difference method. ^3^ Used in the calculation of SID Ca from the feed ingredient in the presence of phytase using the difference method. ^4^ Used in the calculation of SID Ca from the limestone and SID P from the ingredient in the presence of phytase using the difference method. ^5^ Limestone in the diet, where “−” indicates the absence of any added limestone, and “+” indicates the addition of limestone in order to achieve a Ca content of 0.65%. The limestone had a particle size of 509.08 µm and a dynamic solubility, determined as per Kim et al. [16], of 56, 88, and 97% at 5, 15, and 30 min, respectively. ^6^ A novel consensus bacterial 6-phytase variant (Axtra^®^ PHY GOLD (analyzed activity 42,820 FTU/g), Danisco Animal Nutrition & Health, IFF, The Netherlands) was used. ^7^ Refer to Table 1. ^8^ Used as an inert filler (Minema Chemicals (Pty) Ltd., Johannesburg, South Africa). ^9^ Provided per kg of complete diet: vitamin A, 12,000 IU; vitamin D3, 5000 IU; vitamin E, 60 mg; vitamin K3, 2 mg; vitamin B1, 2 mg; vitamin B2, 5 mg; niacin (B3), 50 mg; pantothenic acod (B5), 12 mg; pyridoxine, 3 mg; folic acid, 2 mg; vitamin B12, 0.01 mg; biotin, 0.10 mg. ^10^ Provided per kg of the complete diet: manganese from manganese sulfate, 110 mg; iron from iron sulfate, 41.2 mg; zinc form zinc sulfate, 100 mg; copper from copper sulfate, 10 mg; cobalt from cobalt sulfate, 0.5 mg; iodine from calcium iodate, 2.0 mg; selenium from sodium selenite 0.3 mg. ^11^ A commercial xylanase supplied at 1220 U/kg (Axtra^®^ XB, Danisco Animal Nutrition & Health, IFF, The Netherlands). ^12^ Prepared by mixing 33.33% TiO_2_ and 66.66% corn, after which it was ground three times in a hammer mill to pass through a 1.0 mm. ^13^ Calculated as analyzed phytic acid × 28.18%.

**Table 3:** Dietary formulations of test diets for Experiment 1.

Ingredient	ǀ-------Corn------ǀ		ǀ-----Sorghum-----ǀ		ǀ-------Wheat-------ǀ		ǀ---------FFS ^1^---------ǀ	
LS ^2^	−	+	−	+	−	+	−	+
Phytase ^3^, FTU/kg diet	0/1000	0/1000	0/1000	0/1000	0/1000	0/1000	0/1000	0/1000
Ingredients, %								
Corn, 8% CP	71.15	71.15	−	−	−	−	−	−
Sorghum, 8% CP	−	−	89.53	89.53	−	−	−	−
Wheat, 12% CP	−	−	−	−	51.92	51.92	−	−
FFS, 36% CP	−	−	−	−	−	−	47.12	47.12
Basal ^4^	27.03	27.03	8.68	8.68	46.31	46.31	51.27	51.27
Limestone	−	1.62	−	1.59	−	1.57	−	1.41
Celite ^5^	1.62	−	1.59	−	1.57	−	1.41	−
Vitamin premix ^6^	0.15	0.15	0.15	0.15	0.15	0.15	0.15	0.15
Mineral premix ^7^	0.15	0.15	0.15	0.15	0.15	0.15	0.15	0.15
Salt	0.20	0.20	0.20	0.20	0.20	0.20	0.20	0.20
Sodium bicarbonate	0.20	0.20	0.20	0.20	0.20	0.20	0.20	0.20
Choline chloride, 60%	0.20	0.20	0.20	0.20	0.20	0.20	0.20	0.20
Xylanase ^8^	0.01	0.01	0.01	0.01	0.01	0.01	0.01	0.01
Ti blend ^9^	1.50	1.50	1.50	1.50	1.50	1.50	1.50	1.50
Total	100.00	100.00	100.00	100.00	100.00	100.00	100.00	100.00
Formulated (analyzed values)								
Total Ca	0.03 (0.03)	0.65 (0.67)	0.04 (0.03)	0.65 (0.67)	0.04 (0.05)	0.65 (0.66)	0.12 (0.12)	0.65 (0.67)
Total P	0.17 (0.18)	0.17 (0.17)	0.21 (0.21)	0.21 (0.21)	0.22 (0.24)	0.22 (0.24)	0.21 (0.21)	0.21 (0.23)
Phytate P ^10^	0.14 (0.12)	0.14 (0.12)	0.14 (0.13)	0.14 (0.13)	0.14 (0.14)	0.14 (0.14)	0.14 (0.15)	0.14 (0.15)

^1^ Full-fat soybean meal. ^2^ Limestone in the diet, where “−” indicates the absence of any added limestone, and “+” indicates the addition of limestone in order to achieve a Ca content of 0.65%. The limestone has a particle size of 509.08 µm and a dynamic solubility, determined as per Kim et al. [16], of 56, 88, and 97% at 5, 15, and 30 min, respectively. ^3^ A novel consensus bacterial 6-phytase variant (Axtra^®^ PHY GOLD (analyzed activity 42,820 FTU/g), Danisco Animal Nutrition & Health, IFF, The Netherlands) was used. ^4^ Refer to Table 1. ^5^ Used as an inert filler (Minema Chemicals (Pty) Ltd., South Africa). ^6^ Provided per kg of complete diet: vitamin A, 12,000 IU; vitamin D3, 5000 IU; vitamin E, 60 mg; vitamin K3, 2 mg; vitamin B1, 2 mg; vitamin B2, 5 mg; niacin (B3), 50 mg; pantothenic acod (B5), 12 mg; pyridoxine, 3 mg; folic acid, 2 mg; vitamin B12, 0.01 mg; biotin, 0.10 mg. ^7^ Provided per kg of the complete diet: manganese from manganese sulfate, 110 mg; iron from iron sulfate, 41.2 mg; zinc form zinc sulfate, 100 mg; copper from copper sulfate, 10 mg; cobalt from cobalt sulfate, 0.5 mg; iodine from calcium iodate, 2.0 mg; selenium from sodium selenite 0.3 mg. ^8^ A commercial xylanase supplied at 1220 U/kg (Axtra^®^ XB, Danisco Animal Nutrition & Health, IFF, The Netherlands). ^9^ Prepared by mixing 33.33% TiO_2_ and 66.66% corn after which it was ground three times in a hammer mill to pass through a 1.0 mm. ^10^ Calculated as analyzed phytic acid × 28.18%.

**Table 4:** Formulated and analyzed values for phytase in Experiment 1 diets.

Ingredient	LS ^1^	Formulated Phytase ^2^, FTU/kg Diet	Analyzed Phytase, FTU/kg Diet
Corn	−	0/1000	<100/1020
Corn	+	0/1000	<100/1056
Wheat	−	0/1000	<100/1093
Wheat	+	0/1000	<100/1017
Sorghum	−	0/1000	<100/1094
Sorghum	+	0/1000	<100/1080
FFS ^3^	−	0/1000	<100/1062
FFS	+	0/1000	<100/1100

^1^ “−” indicates the absence of any added limestone, and “+” indicates the addition of limestone in order to achieve a Ca content in the final diet of 0.65%. ^2^ A novel consensus bacterial 6-phytase variant (Axtra^®^ PHY GOLD (analyzed activity 42,820 FTU/g), Danisco Animal Nutrition & Health, IFF, The Netherlands) was used. ^3^ Full-fat soybean meal.

**Table 5:** Dietary formulation of test diets in Experiment 2.

Ingredient	ǀ-------------SBM ^1^-----------ǀ		ǀ-------------RSM ^2^-----------ǀ		ǀ---------SFM ^3^--------ǀ	
LS ^4^	−	+	−	+	−	+
Phytase ^5^, FTU/kg diet	0/1000	0/1000	0/1000	0/1000	0/1000	0/1000
Ingredients, %						
SBM, 46% CP	65.10	65.10	−	−	−	−
RSM, 35% CP	−	−	28.03	28.03	−	−
SFM, 38% CP	−	−	−	−	28.85	28.85
Basal	31.21	31.21	68.42	68.42	67.3	67.3
Limestone	−	1.28	−	1.14	−	1.44
Celite ^6^	1.28	−	1.14	−	1.44	−
Vitamin premix ^7^	0.15	0.15	0.15	0.15	0.15	0.15
Mineral premix ^8^	0.15	0.15	0.15	0.15	0.15	0.15
Salt	0.20	0.20	0.20	0.20	0.20	0.20
Sodium bicarbonate	0.20	0.20	0.20	0.20	0.20	0.20
Choline chloride, 60%	0.20	0.20	0.20	0.20	0.20	0.20
Xylanase ^9^	0.01	0.01	0.01	0.01	0.01	0.01
Ti blend ^10^	1.50	1.50	1.50	1.50	1.50	1.50
Total	100.00	100.00	100.00	100.00	100.00	100.00
Formulated (analyzed values)						
Total Ca	0.19 (0.17)	0.65 (0.66)	0.23 (0.26)	0.65 (0.70)	0.15 (0.12)	0.65 (0.66)
Total P	0.33 (0.36)	0.33 (0.36)	0.31 (0.31)	0.31 (0.33)	0.29 (0.28)	0.29 (0.28)
Phytate P ^11^	0.23 (0.24)	0.23 (0.24)	0.23 (0.20)	0.23 (0.20)	0.23 (0.22)	0.23 (0.22)

^1^ Soybean meal. ^2^ Rapeseed meal. ^3^ Sunflower meal. ^4^ Limestone in the diet, where “−” indicates the absence of any added limestone, and “+” indicates the addition of limestone in order to achieve a Ca content of 0.65%. The limestone has a particle size of 509.08 µm and a dynamic solubility, determined as per Kim et al. [16], of 56, 88, and 97% at 5, 15, and 30 min, respectively. ^5^ A novel consensus bacterial 6-phytase variant (Axtra^®^ PHY GOLD (analyzed activity 42,820 FTU/g), Danisco Animal Nutrition & Health, IFF, The Netherlands) was used. ^6^ Used as an inert filler (Minema Chemicals (Pty) Ltd., South Africa). ^7^ Provided per kg of complete diet: vitamin A, 12,000 IU; vitamin D3, 5000 IU; vitamin E, 60 mg; vitamin K3, 2 mg; vitamin B1, 2 mg; vitamin B2, 5 mg; niacin (B3), 50 mg; pantothenic acod (B5), 12 mg; pyridoxine, 3 mg; folic acid, 2 mg; vitamin B12, 0.01 mg; biotin, 0.10 mg. ^8^ Provided per kg of the complete diet: manganese from manganese sulfate, 110 mg; iron from iron sulfate, 41.2 mg; zinc form zinc sulfate, 100 mg; copper from copper sulfate, 10 mg; cobalt from cobalt sulfate, 0.5 mg; iodine from calcium iodate, 2.0 mg; selenium from sodium selenite 0.3 mg. ^9^ A commercial xylanase supplied at 1220 U/kg (Axtra^®^ XB, Danisco Animal Nutrition & Health, IFF, The Netherlands). ^10^ Prepared by mixing 33.33% TiO_2_ and 66.66% corn after which it was ground three times in a hammer mill to pass through a 1.0 mm. ^11^ Calculated as analyzed phytic acid × 28.18%.

**Table 6:** Formulated and analyzed values for phytase in Experiment 2 diets.

Ingredient	LS ^1^	Formulated Phytase ^2^, FTU/kg Diet	Analyzed Phytase, FTU/kg Diet
SBM ^3^	−	0/1000	<100/1132
SBM	+	0/1000	<100/1060
RSM ^4^	−	0/1000	<100/1097
RSM	+	0/1000	<100/932
SFM ^5^	−	0/1000	<100/1083
SFM	+	0/1000	<100/1091

^1^ “−” indicates the absence of any added limestone, and “+” indicates the addition of limestone in order to achieve a Ca content of the final diet of 0.65%. ^2^ A novel consensus bacterial 6-phytase variant (Axtra^®^ PHY GOLD (analyzed activity 42,820 FTU/g), Danisco Animal Nutrition & Health, IFF, The Netherlands) was used. ^3^ Soybean meal. ^4^ Rapeseed meal. ^5^ Sunflower meal.

**Table 7:** The standardized ileal digestibility (SID) Ca and P of the basal diets in the presence and absence of limestone and phytase.

Diet	LS ^1^	Phytase ^2^, FTU/kg Diet	SID Ca	SID P
Treatment 1	−	0	23.76	63.59
Treatment 2	−	1000	29.30	66.81
Treatment 3	+	0	71.88	48.03
Treatment 4	+	1000	75.20	67.26
SEM ^3^			1.23	0.96

^1^ “−” indicates the absence of any added limestone, and “+” indicates the addition of limestone in order to achieve a Ca content of the final diet of 0.65%. ^2^ A novel consensus bacterial 6-phytase variant (Axtra^®^ PHY GOLD (analyzed activity 42,820 FTU/g), Danisco Animal Nutrition & Health, IFF, The Netherlands) was used. ^3^ Standard error of the mean.

**Table 8:** Standardized ileal digestibility (SID) of Ca and P from different feed ingredients and limestone (LS) inclusions.

Ingredient	LS ^1^	Phytase ^2^, FTU/kg Diet	SID Ca _Ing_^3^	SID P ^4^	SID Ca _LS_^5^
Corn	−	0	46.40 ^d^	74.92 ^bc^	
Corn	−	1000	69.81 ^b^	88.96 ^a^	
Corn	+	0		7.93 ^g^	66.13
Corn	+	1000		64.49 ^d^	69.24
Wheat	−	0	72.87 ^b^	69.79 ^cd^	
Wheat	−	1000	87.32 ^a^	81.64 ^ab^	
Wheat	+	0		33.32 ^f^	53.87
Wheat	+	1000		75.20 ^bc^	64.18
FFS ^6^	−	0	69.85 ^b^	78.85 ^bc^	
FFS	−	1000	72.98 ^ab^	88.78 ^a^	
FFS	+	0		46.54 ^e^	63.76
FFS	+	1000		78.92 ^bc^	67.93
Sorghum	−	0	54.44 ^cd^	78.36 ^bc^	
Sorghum	−	1000	77.57 ^ab^	90.33 ^a^	
Sorghum	+	0		42.97 ^ef^	75.38
Sorghum	+	1000		82.74 ^ab^	83.00
SEM ^7^			3.32	1.59	4.31
Main effects					
Ingredient					
Corn					67.68 ^ab^
Wheat					59.03 ^b^
FFS					65.85 ^b^
Sorghum					79.19 ^a^
SEM					3.16
LS	−				
	+				
	SEM				
Phytase		0			64.78 ^b^
		1000			71.09 ^a^
		SEM			2.19
*p*-values (Ingredient × LS × Phytase) ^8^					
Ingredient			<0.001	<0.01	<0.001
LS				<0.01	
Phytase			<0.001	<0.01	0.048
Ingredient × LS				<0.01	
Ingredient × Phytase			0.02	<0.01	0.856
LS × Phytase				<0.01	
Ingredient × LS × Phytase				<0.01	

^a–g^ Means within a column with different superscripts differ (*p* < 0.05). Main effects are shown only when the interaction was not significant (*p* > 0.05). ^1^ “−” indicates the absence of any added limestone, and “+” indicates the addition of limestone in order to achieve a Ca content in the final diet of 0.65%. ^2^ A novel consensus bacterial 6-phytase variant (Axtra^®^ PHY GOLD (analyzed activity 42,820 FTU/g), Danisco Animal Nutrition & Health, IFF, The Netherlands) was used. ^3^ Standard ileal digestibility of Ca from the ingredient. ^4^ Standard ileal digestibility of P from the ingredient. ^5^ Standard ileal digestibility of Ca from the limestone. ^6^ Full-fat soybean meal. ^7^ Standard error of the mean. ^8^ Where the interaction or effect was not pertinent there was no *p*-value reported.

**Table 9:** Standardized ileal digestibility (SID) of Ca and P from different feed ingredients and limestone (LS) inclusions.

Ingredient	LS ^1^	Phytase ^2^, FTU/kg	SID Ca _Ing_^3^	SID P ^4^	SID Ca _LS_^5^
SBM ^6^	−	0	46.80 ^b^	81.59 ^bc^	
SBM	−	1000	67.77 ^a^	89.86 ^a^	
SBM	+	0		49.11 ^f^	37.76 ^c^
SBM	+	1000		77.51 ^c^	56.64 ^ab^
RSM ^7^	−	0	42.65 ^b^	57.88 ^e^	
RSM	−	1000	62.32 ^a^	81.70 ^bc^	
RSM	+	0		31.99 ^g^	39.73 ^c^
RSM	+	1000		59.04 ^de^	56.26 ^ab^
SFM ^8^	−	0	60.99 ^a^	74.93 ^c^	
SFM	−	1000	61.65 ^a^	85.94 ^ab^	
SFM	+	0		33.75 ^g^	52.51 ^b^
SFM	+	1000		65.69 ^d^	61.02 ^a^
SEM ^9^			2.35	1.60	2.42
*p*-values (Ingredient × LS × Phytase) ^10^					
Ingredient			0.01	<0.001	<0.001
LS				<0.001	
Phytase			<0.001	<0.001	0.09
Ingredient × LS			<0.001		
Ingredient × Phytase			<0.001	0.014	<0.001
LS × Phytase				<0.001	
Ingredient × LS × Phytase				<0.001	

^a–g^ Means within a column with different superscripts differ (*p* < 0.05). Main effects are shown only when the interaction is not significant (*p* > 0.05). ^1^ Limestone in the diet, where “−” indicates the absence of any added limestone, and “+” indicates the addition of limestone in order to achieve a Ca content of 0.65%. ^2^ A novel consensus bacterial 6-phytase variant (Axtra^®^ PHY GOLD (analyzed activity 42,820 FTU/g), Danisco Animal Nutrition & Health, IFF, The Netherlands) was used. ^3^ Standard ileal digestibility of Ca from the ingredient. ^4^ Standard ileal digestibility of P from the ingredient. ^5^ Standard ileal digestibility of Ca from the limestone. ^6^ Soybean meal. ^7^ Rapeseed meal. ^8^ Sunflower meal. ^9^ Standard error of the mean. ^10^ The analysis of the interactions and effects were reported, however in the case where the interaction or effect was not explored there was no *p*-value reported.

## Data Availability

Data are unavailable due to privacy or ethical restrictions by the institutions.
